# Impact of Switching Between Different Brands of Levothyroxine on Maintaining Normal Thyrotropin Levels: A Prospective, Multicenter, Phase IV Clinical Trial

**DOI:** 10.7759/cureus.112775

**Published:** 2026-07-16

**Authors:** Banshi Saboo, Parag Shah, Ramesh Goyal, Vivek Arya, Kandarp Bhuva, Dharmendra Panchal, Vishal Dave, Vivek Sharma, Deepak Bunger, Alok Chaturvedi

**Affiliations:** 1 Endocrinology, Diabetes Care and Hormone Clinic, Ahmedabad, IND; 2 Endocrinology, Gujarat Endocrine Centre, Ahmedabad, IND; 3 Endocrinology, Gujarat Super Specialty Clinic, Ahmedabad, IND; 4 Endocrinology and Diabetes, Center for Endocrine Disease and Diabetes, Ahmedabad, IND; 5 General Medicine, Galaxy Hospital, Ahmedabad, IND; 6 Endocrinology and Diabetes, Diabetes Care and Hormone Clinic, Ahmedabad, IND; 7 Medical Affairs, Intas Pharmaceuticals Limited, Ahmedabad, IND

**Keywords:** eltroxin, hypothyroidism, lethyrox, levothyroxine, tsh

## Abstract

Background

This study aimed to evaluate the impact of a switch from Thyronorm or Eltroxin tablets to Lethyrox tablets on serum thyroid-stimulating hormone (TSH) levels in participants with primary hypothyroidism who were stable on Thyronorm or Eltroxin.

Methodology

Patients aged 18 to 65 years diagnosed with primary hypothyroidism for at least a year, who were taking a stable dose of levothyroxine tablets (Thyronorm or Eltroxin) for at least the last eight weeks and had normal serum TSH levels (0.465 to 4.68 mIU/L), were included in this prospective, multicenter, phase IV clinical trial. For a period of 18 weeks, the patients were transferred to Lethyrox tablets at the same dosage they had previously been taking. The percentage of patients with normal blood TSH levels after six weeks of switching was the main study objective.

Results

A total of 150 patients were enrolled, with a mean (SD) age of 45 (11) years, including 104 (69.3%) females. The mean (SD) serum TSH at baseline was 2.49 (1.12) mIU/L. Overall, 142 patients qualified for the intent-to-treat analysis. Of these, 111 (78.2%) patients maintained normal serum TSH values after six weeks of switching to Lethyrox tablet. The serum TSH level was within the normal reference range for 104 (73.2%) patients and 108 (76.1%) patients after 12 and 18 weeks of switching, respectively. A dose change was not required in 129 (90.84%) patients after switching to Lethyrox tablet at weeks 6 and 12, respectively. The mean (SD) TSH values at 6, 12, and 18 weeks were 3.55 (7.90), 3.71 (4.83), and 3.00 (2.03) mIU/L, respectively.

Conclusions

In the majority of individuals with primary hypothyroidism, switching brands of levothyroxine tablets was safe as it did not result in clinically significant changes in serum TSH levels.

## Introduction

Hypothyroidism is characterized by a wide range of clinical manifestations, including asymptomatic, overt myxoedema and multisystem failure [1]. In the developed world, hypothyroidism affects 4-5% of people [2] and subclinical hypothyroidism affects 4-15% of people [3]. The prevalence rate of hypothyroidism in India is around 11% [4]. To manage thyroid-related symptoms, hypothyroidism patients require lifelong replacement treatment with levothyroxine (LT4) to attain normal serum thyroid-stimulating hormone (TSH) levels.

Levothyroxine was first developed in 1927 [5], but its patent was not granted to any organization. Synthyroid is one of the most commonly used branded generic formulations of levothyroxine that has been available in the US market since the 1950s, even though it received US Food and Drug Administration (FDA) approval in 2002 [6]. In India, Eltroxin and Thyronorm are two of the most commonly prescribed brands. Levothyroxine sodium is one of the most commonly prescribed drugs in the United States, with approximately 25 million individuals using levothyroxine in 2016 [1,3]. Levothyroxine is available as a generic or brand-name product. The US FDA has determined that generic levothyroxine formulations are bioequivalent to brand-name reference-listed drugs [7]. However, generic levothyroxine has been less widely prescribed than other generic pharmaceutical products [3,7]. One potential reason for this may be lingering concerns about an association between switching levothyroxine products sourced from different manufacturers and the stability of thyroid hormone levels [4,5].

In particular, the 2014 American Thyroid Association recommendation advises against switching between levothyroxine formulations that are derived from different manufacturers due to concerns with the FDA’s proposed bioequivalence techniques [5]. The recommendation advises physicians to prescribe either a particular brand of levothyroxine or the same identified generic levothyroxine product (i.e., derived from the same manufacturer) to preserve consistency in the levothyroxine product. The guidelines suggest that dispensing generic levothyroxine may be linked to changes in thyroid hormone levels or amounts needed because the latter approach is logistically simpler. The FDA’s stance that authorized generic levothyroxine medications should be interchangeable without requiring serum thyrotropin (TSH) testing conflicts with this guideline advice [6].

Lethyrox (levothyroxine tablets, manufactured by Intas Pharmaceuticals Ltd.) was approved in India in 2017 for the management of hypothyroidism. Levothyroxine manufactured by Intas Pharmaceuticals Ltd. is also approved in the United States since October 2020 and can be switched with Synthyroid, Levoxyl, Thyro-tabs, and Unithroid tablets based on successful bioequivalence studies. However, real-world data on the clinical usage of Lethyrox is not available. Hence, this phase IV trial was conducted to determine the efficacy and safety of a dose-equivalent levothyroxine brand switch in patients with primary hypothyroidism who were receiving a stable dose of Thyronorm or Eltroxin.

This article was previously presented as a poster at the 2024 American Association of Clinical Endocrinology Annual Scientific Meeting in May 2024.

## Materials and methods

This prospective, interventional, open-label, single-arm, phase IV clinical trial was conducted at six trial centers in India and enrolled patients from May 2022 to May 2023. The trial was registered at the Clinical Trials Registry-India (CTRI) (registration number CTRI/2022/05/042396). The study protocol and related documents were reviewed and approved by the institutional review boards (IRBs) or independent ethics committees (IECs). The study was conducted in accordance with the ethical principles that have their origin in the Declaration of Helsinki [7] and in accordance with the International Conference on Harmonization’s Good Clinical Practice (ICH-GCP) guidelines [8], New Drugs and Clinical Trial (NDCT) Rules 2019 [9], Indian Council of Medical Research guidelines (2017) [10], applicable regulatory requirements, and in compliance with the study protocol. All participants provided written informed consent to participate in the study. The reporting of our study adhered rigorously to the guidelines set forth by Equator Network [11], following the CONSORT [12] statement, which ensures transparency and completeness in reporting the research.

Participants

Patients aged 18-65 years (both inclusive) of either sex, diagnosed with primary hypothyroidism for at least one year from the screening visit, were eligible for study participation. Patients were included if they were stabilized on the same morning dose of Thyronorm or Eltroxin (a single daily tablet at either of following dose strengths only: 25 μg, 50 μg, 75 μg, 88 μg, 100 μg, 125 μg, 150 μg) for at least last eight weeks before the screening visit and had serum TSH levels within the normal reference range (0.465-4.68 mIU/L). Patients were excluded if they had a major surgery within eight weeks before screening, were diagnosed with panhypopituitarism or secondary hypothyroidism and a medical condition (documented medical history of clinically significant cardiac, renal, vascular, pulmonary, gastrointestinal, endocrine, neurologic, hematologic, rheumatologic, psychiatric, metabolic disturbances, or any other medical condition(s) for which, in the opinion of the investigator, participation would not be in the best interest of the participant or that could prevent, limit, or confound the protocol-specified assessments), or used medications that could affect thyrotropin levels. Additionally, patients with known allergies, hypersensitivity, or intolerance to Lethyrox tablet or its excipients were also excluded.

Treatment

The eligible patients received Lethyrox tablets orally once daily (same dose as that of Thyronorm or Eltroxin taken earlier) in the morning on an empty stomach from baseline to the end of week 18. All patient received a single tablet of 25 μg/50 μg/75 μg/88 μg/100 μg/125 μg/150 μg as per their pre-study levothyroxine regimen (Thyronorm or Eltroxin).

Study design and sample size

**Figure 1:**
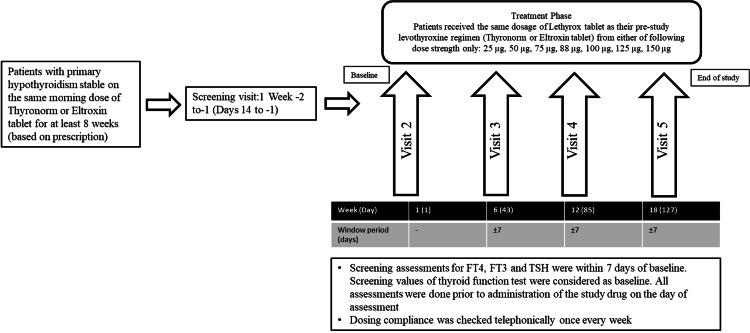
Study design. TSH = thyroid-stimulating hormone; FT3 = free triiodothyronine; FT4 = free thyroxine

To prove the suitability of switching between the levothyroxine brands and to generate evidence data after the switch to the levothyroxine brand, this phase 4 study was planned. Considering the switch from Thyronorm or Eltroxin Tablet to Lethyrox Tablet, a single-arm design with a test arm was selected. The study was conducted in two phases: a two-week screening phase, intervention phase extending from day 1 (baseline) to the end of week 18, as shown in Figure 1. The duration of individual participation was approximately 20 weeks, and the treatment duration was 18 weeks. Considering a phase 4 study and a non-comparative study design, no formal power-based sample size calculation was conducted. Hence, 120 participants were assumed to be sufficient to meet the study objective. Considering 20% dropouts and/or withdrawals, 150 participants were enrolled.

Study assessments

The primary endpoint was the proportion of patients who maintained normal serum TSH levels after six weeks of the switch. The secondary endpoints included the proportion of patients who maintained normal serum TSH values after 12 and 18 weeks of the switch; absolute and relative percent change from baseline of serum TSH values after switch at 6 weeks, 12 weeks, and 18 weeks; and change from baseline of serum free thyroxine (FT4), serum free triiodothyronine (FT3) after switch at 6, 12, and 18 weeks. Additional secondary endpoints included the proportion of patients who did not need a change of dose after the switch at 6, 12, and 18 weeks, and the magnitude of the change in daily dose needed after the switch at 6, 12, and 18 weeks (the magnitude was determined via a change table which provided the proportion of participants that needed a change in daily dose (μg/day) of -25 μg, -12.5 μg, -6.25 μg, 0 μg, or +12.5 μg). Safety and tolerability were assessed via adverse events reported after the switch from Thyronorm/ Eltroxin tablet to Lethyrox tablet in patients who were stable on Thyronorm or Eltroxin.

Statistical analysis

The safety and efficacy were evaluated in safety, intent-to-treat (ITT), and per-protocol (PP) sets. The safety set was defined as all enrolled patients who received at least one dose of study medication. The ITT set included all enrolled patients who had received at least one dose of the trial medication and had undergone at least one efficacy evaluation. The PP set included all enrolled patients who completed the study with no major protocol deviation. Lethyrox tablets were administered once daily for 18 weeks. The treatment phase was considered from day 1 (baseline) to the end of week 18. All statistical analyses were performed using SAS® Version 9.4 (SAS Institute Inc., Cary, NC, USA). All statistical testing was two-sided at an α of 0.05, if not specified. Baseline and demographic characteristics were summarized using descriptive statistics. Continuous variables were summarized using summary statistics (number of observations, mean, standard deviation (SD), median, minimum, and maximum values, etc.), as applicable. Categorical values were summarized using frequencies and percentages.

## Results

Patient disposition

**Figure 2:**
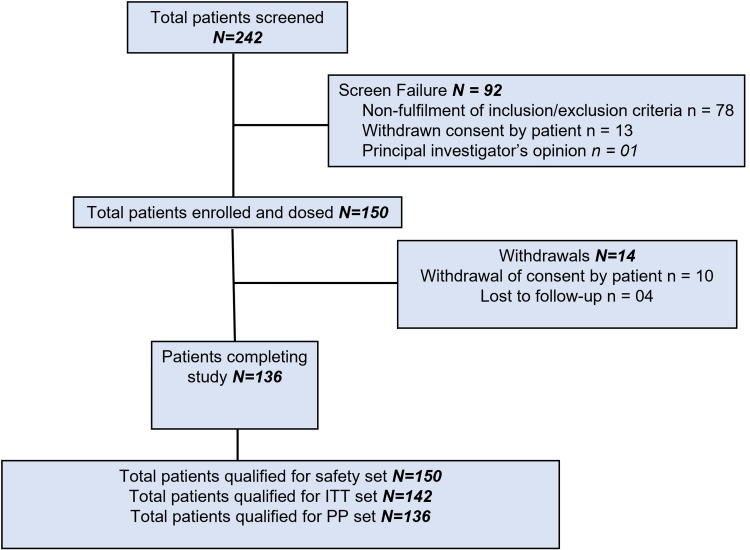
Patient disposition. Safety set: defined as all enrolled patients who received at least one dose of trial medication. Intent-to-treat (ITT) set: defined as all enrolled patients who received at least one dose of trial medication and underwent at least one efficacy evaluation. Per protocol (PP) set: defined as all enrolled patients who completed the trial with no major protocol deviations.

A total of 242 patients were screened, of whom 150 patients were included in the study. In total, 136 patients successfully completed the trial, while 14 patients were withdrawn from the trial (Figure 2). The mean (SD) age of the patients was 45 (11) years, and the mean (SD) weight was 70.4 ± 13.03 kg. The racial makeup of the trial was 100% Asian (safety set). The majority of the patients were females (104, 69.3%), the mean (SD) body mass index was 28.4 (5.4) kg/m², and the mean (SD) serum TSH was 2.49 (1.12) mIU/L (Table 1).

**Table 1:** Baseline characteristics. BMI = body mass index; TSH = thyroid-stimulating hormone

Characteristics	N = 150
Age (years), mean (SD)	45 (11)
Gender, n (%)	
Male	46 (30.7)
Female	104 (69.3)
BMI (kg/m²), mean (SD)	28.4 (5.4)
Serum TSH (mIU/L), mean (SD)	2.49 (1.12)

Efficacy results

**Figure 3:**
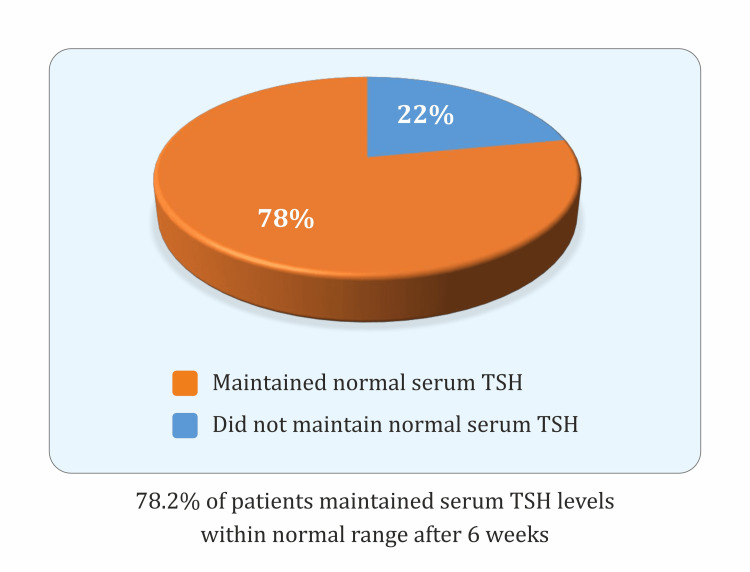
Proportion of patients who maintained serum TSH levels within the normal reference range after 6 weeks of switch. TSH = thyroid-stimulating hormone

**Figure 4:**
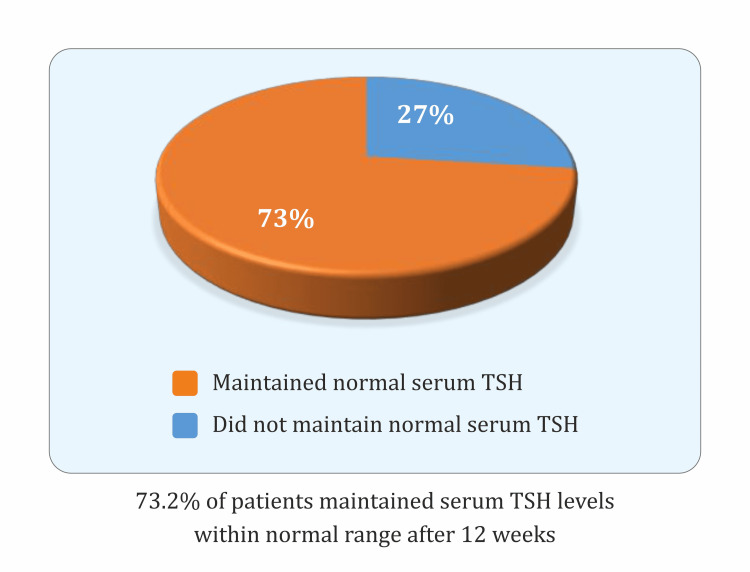
Proportion of patients who maintained serum TSH levels within the normal reference range after 12 weeks of switch. TSH = thyroid-stimulating hormone

**Figure 5:**
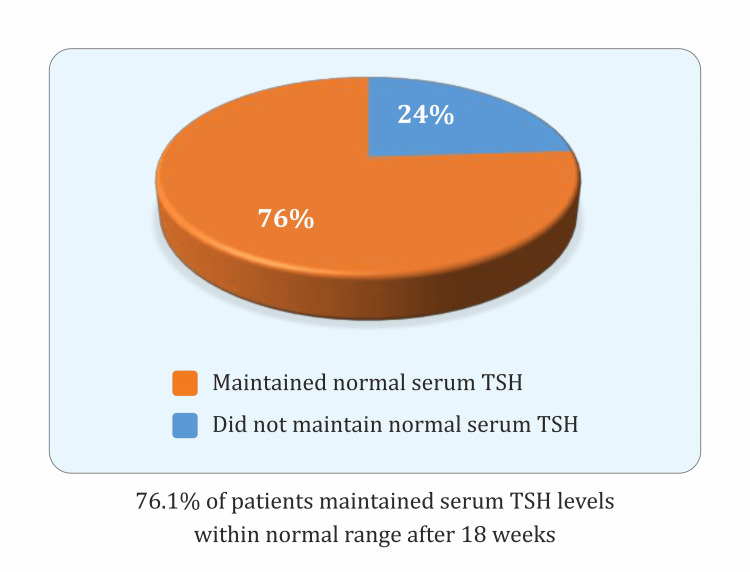
Proportion of patients who maintained serum TSH levels within the normal reference range after 18 weeks of switch. TSH = thyroid-stimulating hormone

**Figure 6:**
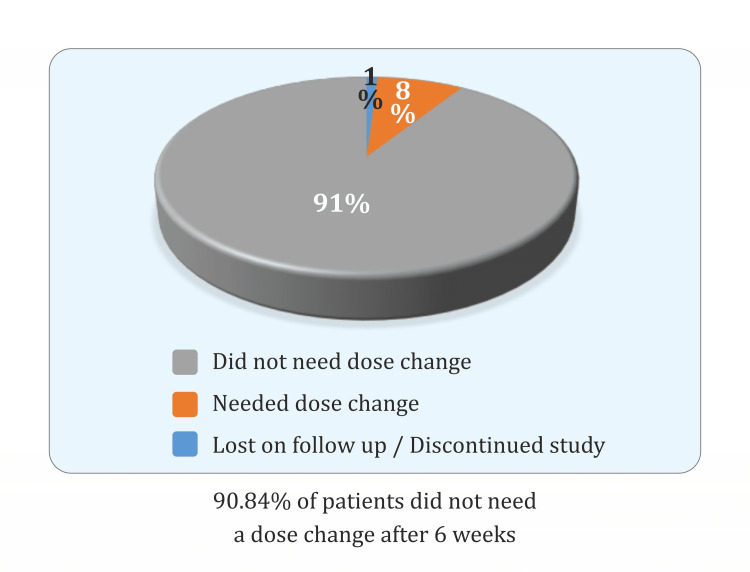
Proportion of patients who did not need change in the dose after 6 weeks of switch.

**Figure 7:**
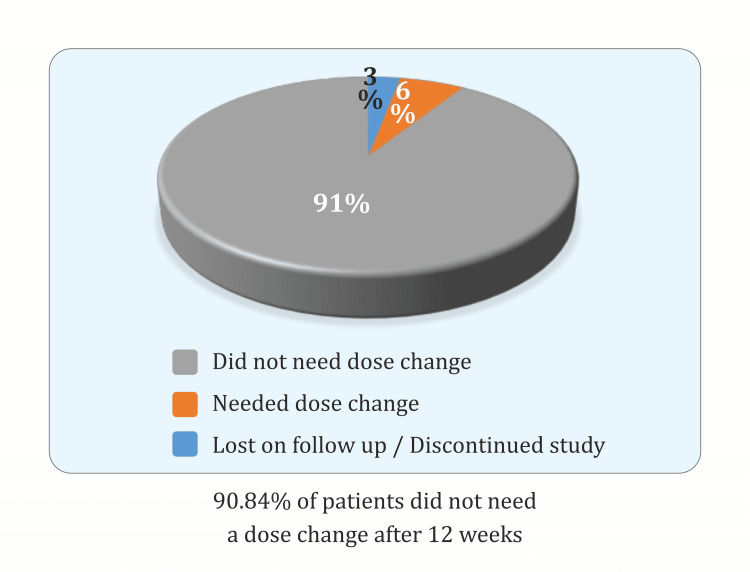
Proportion of patients who did not need change in the dose after 12 weeks of switch.

A total of 150 patients were enrolled in the study, and among them, 142 and 136 patients qualified for the ITT and PP sets, respectively. The serum TSH was within the normal reference range for 111 (78.2%) patients after the switch at six weeks (Figure 3). The mean TSH value observed after 12 weeks was 3.71 ± 4.832 (p = 0.0047) in the ITT set. The study generated statistically significant evidence after 12 weeks of treatment with Lethyrox in maintaining TSH levels. Overall, 104 (73.2%) patients obtained normal serum TSH (mIU/L) after 12 weeks of the switch (Figure 4). The mean serum TSH value observed after 18 weeks was 3.0 ± 2.035 (p = 0.0079). The proportion of patients who obtained normal serum TSH (mIU/L) after switching at 18 weeks was 108 (76.1%) (Figure 5). The mean (SD) absolute change from the baseline of serum TSH values after switch at 6, 12, and 18 weeks in the ITT set was 1.01 (8.1), 1.23 (5.0), and 0.52 (2.24) mIU/L, respectively. The mean (SD) relative percentage change from baseline in serum FT3 values after switch at 6, 12, and 18 weeks in the ITT set was 4.01 (21.86), 0.82 (22.4), and -1.0 (18.9), respectively, as shown in Table 2. The mean (SD) relative percentage change from baseline in serum FT4 values after switch at 6, 12, and 18 weeks in the ITT set was 0.76 (18.8), 4.56 (21.23), and 4.74 (20.37), respectively. Overall, 129 (90.84%) patients did not need a change in the levothyroxine dose after both 6 and 12 weeks of the switch, respectively (Figures 6, 7). There were no adverse events or serious adverse events reported in the study.

**Table 2:** TSH, FT3, and FT4 values from baseline to 6, 12, and 18 weeks of treatment. TSH = thyroid-stimulating hormone; FT3 = free triiodothyronine; FT4 = free thyroxine; ITT = intention to treat

ITT set (N = 142)					
		Screening	6 weeks	12 weeks	18 weeks
TSH (mIU/L)	Mean (SD)	2.52 (1.12)	3.55 (7.90)	3.71 (4.83)	3.00 (2.03)
	Median (range)	2.38 (0.01–4.73)	2.50 (0.07–92.9)	2.50 (0.17–39.73)	2.43 (0.10–13.34)
FT3 (pmol/L)	Mean (SD)	4.54 (0.92)	4.62 (0.96)	4.49 (0.86)	4.41 (0.70)
	Median (range)	4.35 (2.89–7.17)	4.38 (1.99–8.03)	4.47 (2.14–6.66)	4.32 (2.56–6.53)
FT4 (pmol/L)	Mean (SD)	13.66 (2.71)	13.52 (2.62)	13.97 (2.67)	14.09 (2.88)
	Median (range)	13.15 (7.70–24.60)	13.10 (6.70–23.40)	13.50 (7.70–23.60)	13.55 (7.48–21.90)

## Discussion

Hypothyroidism is a common condition of thyroid hormone deficiency, which is readily diagnosed and managed but may be potentially fatal in severe cases if untreated. Hypothyroidism is mainly treated with levothyroxine monotherapy. There have been concerns over the switch of levothyroxine products from different manufacturers regarding the stability of thyroid hormone levels [13]. The generic preparations of levothyroxine are relatively less expensive and have been rated by the US FDA as bioequivalent to their brand-name reference list drugs based on successful bioequivalence studies [14]. Physicians are skeptical regarding the comparable efficacy of generic and brand-name levothyroxine as one of the factors contributing to the ongoing preference for brand-name levothyroxine. Three endocrine professional groups issued a united position statement in 2004 questioning the US FDA’s bioequivalence determination process after the FDA authorized the use of many generic levothyroxine products. The position statement made it clear that the US FDA’s approach does not depend on the amount of thyrotropin and does not take into account the function of endogenously generated hormone. They also suggested that there could be a substantial variation in the bioequivalence of generic and brand-name levothyroxine as a result of these methodological issues and the drug’s very limited therapeutic index. This variation may have an impact on thyrotropin levels and related clinical results. The US FDA revised the potency requirements for levothyroxine in 2007 to fulfil a 95% to 105% potency standard until its expiration date in response to concerns expressed by patients and healthcare professionals regarding levothyroxine products [15].

Despite these modifications, the issues brought up by endocrine societies in 2004 still influence the recommendations and practices of endocrine guidelines. For example, switching between levothyroxine formulations is expressly discouraged by the 2014 American Thyroid Association recommendation. The guidelines advise doctors to prescribe either a particular brand-name levothyroxine or the same recognizable formulation of generic levothyroxine to retain the same preparation. The latter approach is simpler logistically as it prevents the pharmacy from substituting another levothyroxine formulation, which frequently occurs when a generic prescription is utilized and may occur without a doctor’s consent [15].

The purpose of our study was to evaluate the safety and efficacy of switching to Lethyrox from Thyronorm or Eltroxin, the most commonly used brands in India. In our study, after brand switching, in the majority of patients (111 (78.2%) in the ITT set), serum TSH levels were found to be within the normal range at six weeks. After 12 weeks and 18 weeks of treatment, 104 (73.2%) and 108 (76.1%) patients maintained normal serum TSH levels, respectively, in the ITT set. Therefore, a large proportion of patients had stable thyroid hormone levels in our study. In a large real-world, retrospective study conducted by Brito et al. [13] in 15,829 patients to evaluate the association between generic-to-generic levothyroxine switching and serum TSH levels, it was found that normal serum TSH levels were maintained in 84.5% among switchers versus 82.7% among non-switchers [14]. Our study findings are very similar to this study, as approximately 80% of the patients maintained normal serum TSH levels irrespective of the fact that they switched the levothyroxine brand. The mean (SD) absolute change in serum TSH values from baseline at 6, 12, and 18 weeks of switch was 1.03 (8.1), 1.19 (5.02), and 0.48 (2.24) mIU/L, respectively. This also shows that the mean change in serum TSH values remained within normal limits. Our study also provides profound evidence in favor of not requiring a dose change of levothyroxine after switching the brand. During our study, dose change was not required in 129 (90.84%) patients after the switch at week 6 and at week 12.

In our study, none of the patients reported any adverse event after the switch of the brand to Lethyrox. One of the possible reasons for the absence of any adverse event in our study could be the relatively small change in serum TSH levels. This reinforces the hypothesis that a switch from Thyronorm or Eltroxin tablet to Lethyrox tablet is feasible and without any compromise with patient safety.

Limitations

This study was conducted only in an Indian population, and it may not be representative of other geographies or races. A large, phase 3, randomized controlled study should be conducted to study the impact of the levothyroxine brand switch on patient safety and serum thyrotropin levels.

## Conclusions

Switching among different brands of levothyroxine tablets was safe and was not associated with clinically significant changes in serum TSH levels in the majority of patients with primary hypothyroidism. The study also provides profound evidence of not requiring a dose change after switching brands. These findings may reassure patients and clinicians that switching between levothyroxine products is unlikely to have a significant impact on treatment outcomes.
